# Severe Malaria Due to Imported Plasmodium ovale: A Case Report From New York

**DOI:** 10.7759/cureus.75650

**Published:** 2024-12-13

**Authors:** Farshad Bagheri, Uchenna Chinakwe, Bilal Jawed, Amber Ehsan Faquih, Hamayl Zeeshan

**Affiliations:** 1 Infectious Disease, Jamaica Hospital Medical Center, Queens, USA; 2 Internal Medicine, Jamaica Hospital Medical Center, Queens, USA; 3 Internal Medicine, Jinnah Postgraduate Medical Centre, Karachi, PAK; 4 Infectious Diseases, University of Alabama at Birmingham, Birmingham, USA; 5 Internal Medicine, Dow University of Health Sciences, Civil Hospital Karachi, Karachi, PAK

**Keywords:** endemic malaria, hypnozoite, imported malaria, : malaria, malaria treatment, medical intensive care unit (micu), plasmodium ovale, primaquine, septic shock [ss], travel history

## Abstract

Malaria is highly prevalent in West and Central Africa. In the United States, most reported cases are due to immigration from endemic regions. Severe malaria caused by Plasmodium ovale is rare. This case report details a young, previously healthy male traveler from North Central Africa who presented to the emergency department with septic shock, later diagnosed as P. ovale malaria. This report aims to raise awareness among healthcare workers about the potential for P. ovale to cause severe malaria and to emphasize the importance of prompt diagnosis and treatment.

## Introduction

Malaria is a parasitic infection caused by species of the genus Plasmodium. One of these species, Plasmodium ovale is responsible for tertian malaria in humans. It is mainly found in tropical West Africa, but it can also be found in the Philippines, Indonesia, and Papua New Guinea, where it remains relatively rare [1]. The cases of malaria that are acquired in endemic areas and subsequently diagnosed and treated in non-endemic countries are referred to as imported malaria. Approximately 70% of the global burden of imported malaria occurs in Europe, while the United States accounts for about 15%. Other countries reporting cases include Australia, Bahrain, Singapore, and Qatar. China has also documented instances of imported malaria, mainly linked to work-related travel from Africa and Asia [2]. The most common species of imported malaria are Plasmodium falciparum and Plasmodium vivax. Data from the United States in 2018 indicated that the predominant species of imported malaria were P. falciparum (69.8%), P. vivax (9.5%), P. ovale (5.2%), and P. malariae (2.6%). Literature indicates that frequent travel to high-risk areas, especially by immigrants visiting family, combined with limited use of preventive measures due to low perceived risk, poses substantial challenges to malaria elimination. These factors, along with the reintroduction of the parasite into malaria-free regions through imported cases, underscore the need for strong surveillance, effective travel health guidance, and community engagement to prevent local transmission and maintain malaria-free status globally [3].

The life cycle of Plasmodium ovale begins when an infected female Anopheles mosquito injects sporozoites into a human host. These sporozoites travel through the bloodstream to the liver within minutes, where they invade liver cells and multiply, forming thousands of merozoites. P. ovale has an incubation period ranging from 12 to 18 days but can extend up to four years due to the presence of hypnozoites-dormant liver-stage forms that can reactivate long after initial infection, causing relapsing infections. After development in the liver, merozoites enter the bloodstream, invade red blood cells, and undergo the intraerythrocytic developmental cycle, which includes stages of growth and division. This asexual cycle takes approximately 48 hours, producing new merozoites that infect additional red blood cells, leading to malaria symptoms such as fever and anemia. Some parasites transition to sexual forms known as gametocytes, which circulate in the blood and are ingested by another mosquito during a blood meal. In the mosquito’s gut, male and female gametocytes mature into gametes, fuse to form a zygote and develop into an ookinete, which penetrates the gut wall and encysts as an oocyst. The oocyst releases sporozoites that migrate to the mosquito’s salivary glands, preparing to infect a new human host and thus completing the life cycle of P. ovale [4,5].

## Case presentation

**Figure 1:**
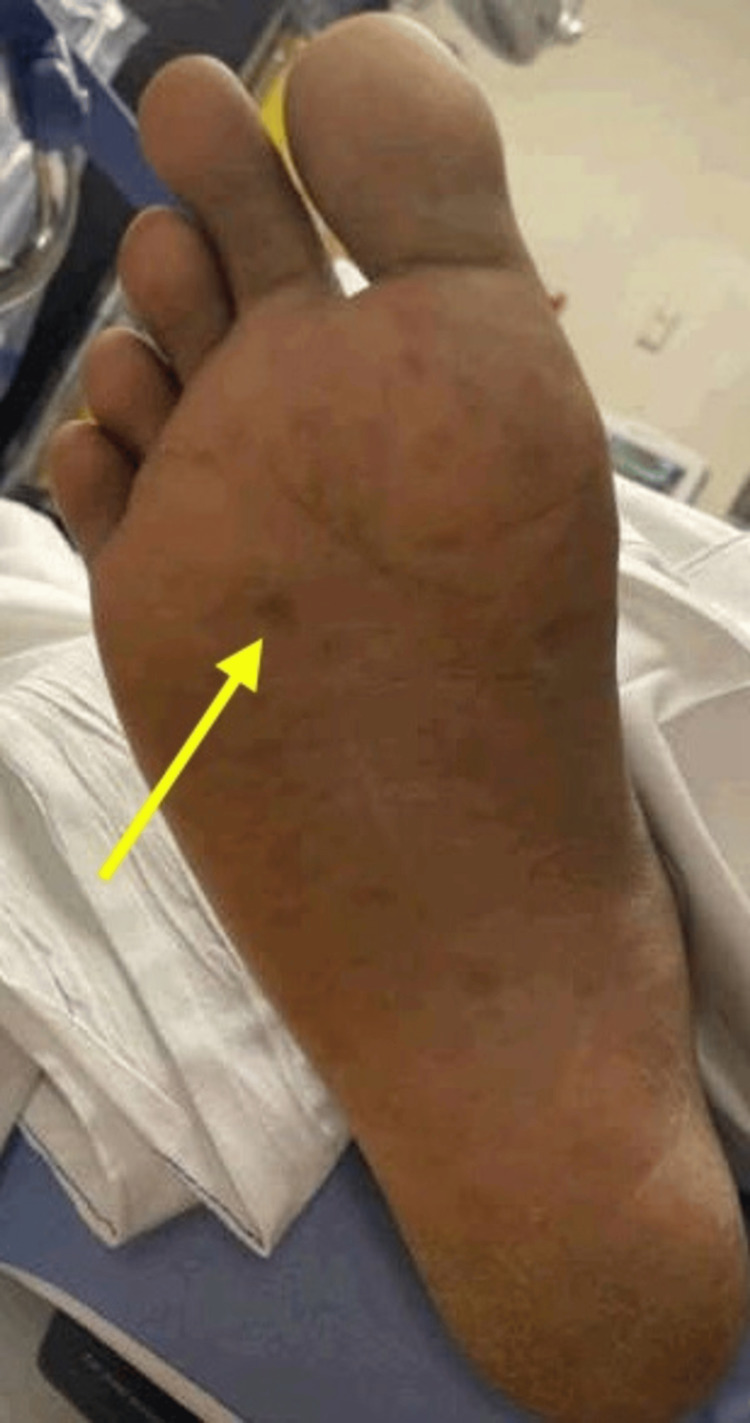
Picture of Ventral side of Right Foot There is a pigmented rash scattered across the sole of the right foot.

**Figure 2:**
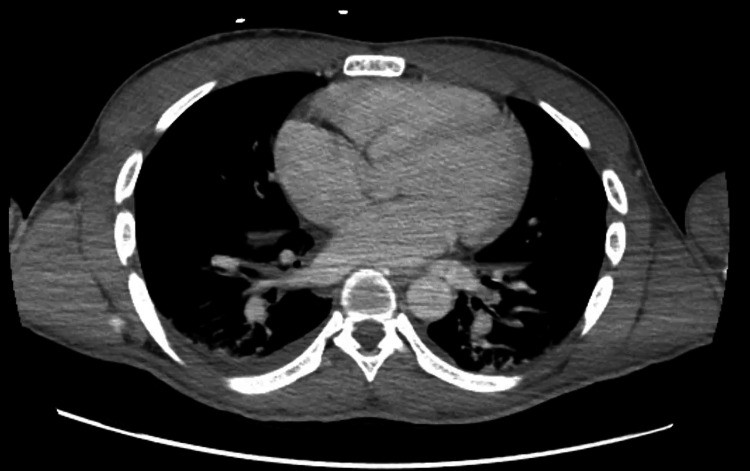
CT scan of the chest This image shows interstitial edema in both lungs and bilateral pleural effusions.

**Figure 3:**
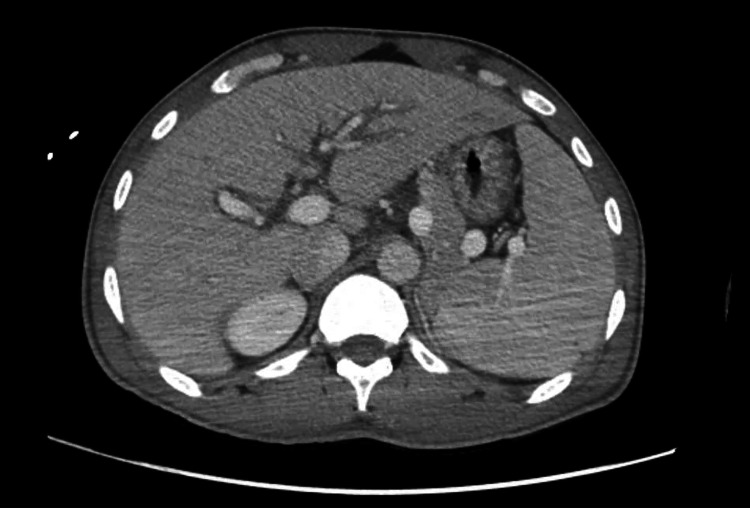
CT scan of the Abdomen and Pelvis This image depicts slight ascites, periportal edema in the liver, pericholecystic edema, hepatomegaly, and splenomegaly.

**Figure 4:**
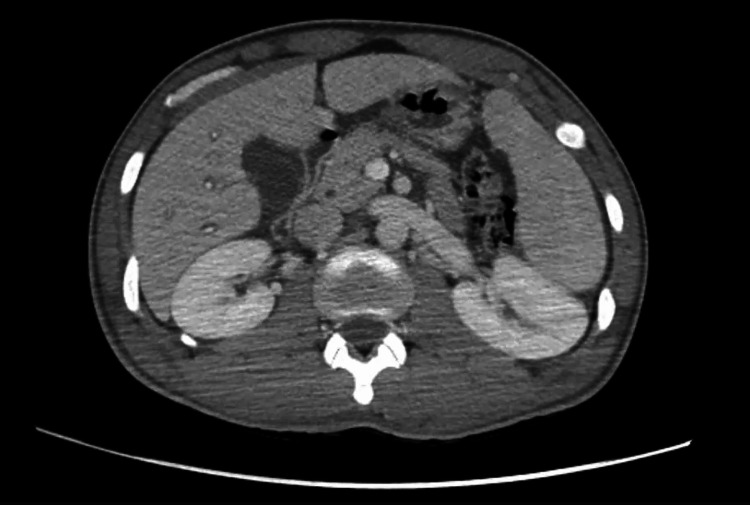
CT scan of the abdomen and pelvis This image depicts generalized edema, slight ascites, periportal edema in the liver, pericholecystic edema, hepatomegaly, and splenomegaly.

A 21-year-old male from Chad in North-Central Africa presented to the Emergency Department (ED) via Emergency Medical Services from the International Airport after an episode of presyncope. The patient had no significant past medical history and reported symptoms of intermittent fever, chills, poor oral intake, and dizziness over the preceding 10 days. He had recently traveled to New York from Chad through Mexico and San Diego over the preceding two months. Upon examination in ED, the patient appeared ill and febrile, with an oral temperature of 102.9°F. His blood pressure was 104/52 mmHg, and his pulse rate was 89 beats per minute. A bilaterally scattered pigmented rash was observed on the palms and soles (Figure 1). Other physical findings, including those from the abdominal, cardiovascular, respiratory, and nervous systems, were unremarkable. The initial lab workup is shown in Table 1. Investigations included a CT scan of the abdomen, pelvis, and chest, revealing generalized edema, interstitial edema in the lungs bilaterally, bilateral pleural effusions, slight ascites, periportal edema in the liver, pericholecystic edema, hepatomegaly, and splenomegaly (Figures 2, 3, 4). While in the ED, the patient became hypotensive and bradycardic, eventually requiring vasopressor support after persistent hypotension despite the administration of 3.5 liters of intravenous crystalloids. He was also started on vancomycin and piperacillin-tazobactam, with blood cultures and other labwork ordered. He was admitted to the Medical Intensive Care Unit. Based on the initial assessment and available lab results, the differential diagnoses included malaria, bacteremia, chikungunya, dengue, syphilis, Coxsackie virus, Rocky Mountain spotted fever, pneumonia, urinary tract infection, and hepatitis C. Infectious disease specialists were consulted for further evaluation.

**Table 1:** Labs on initial presentation The lab results are measured in cells per liter (109/L10^9/L109/L and 1012/L10^{12}/L1012/L), grams per deciliter (g/dL), percentage (%), femtoliters (fL), milliequivalents per liter (mEq/L), milligrams per deciliter (mg/dL), units per liter (U/L), millimoles per liter (mmol/L), nanograms per milliliter (ng/mL), femtoliters (fL) and milligrams per liter (mg/L). MCV: mean corpuscular volume, MCHC: mean corpuscular hemoglobin concentration, RDW: red cell distribution width, MPV: mean platelet volume, BUN: blood urea nitrogen, ALT: alanine aminotransferase, AST: aspartate aminotransferase

Lab test	Result	Normal range
white blood cells	6.6 x 10^9^/L	4.5-11.0 x 10^9^/L
red blood cells	2.61 x 10^12^/L	Male: 4.3-5.9 x 10^12^/L Female: 3.5-5.5 x 10^12^/L
Hemoglobin	7.8 g/dL	Male: 14 to 18 g/dl Female: 12 to 16 g/dl
Hematocrit	23.5 %	Men: 40–54% Women: 36–48%
MCV	90 fL	80-100 fl
MCHC	33.2 g/dL	32–36 g/dL
RDW	13.4 %	Male: 11.8–14.5% Female: 12.2–16.1%
MPV	8.2 fL	8–12 fL
Platelet Count	189 x 10^9^//L	150-400 x 10^9^/L
Neutrophils	78%	40- 60%
lymphocytes	9.9%	20- 40%
monocytes	10.5%	2- 8%
Eosinophils	0.2%	1- 4%
basophils	0.5%	0.5- 1%
glucose	99 mg/dL	70–100 mg/dL
BUN	15mg/dL	8–20 mg/dL
creatinine	0.9mg/dL	Female: 0.50–1.10 mg/dL; male: 0.70–1.30 mg/dL
sodium	129 mEq/L	136–145 mEq/L
potassium	3.4 mEq/L	3.5–5.0 mEq/L
chloride	98 mEq/L	98–106 mEq/L
carbon dioxide	25 mEq/L	23–30 mEq/L
calcium	7.3 mg/dL	8.6–10.2 mg/dL
Anion gap	6	7–13 mEq/L
Phosphorous	2.5mg/dL	3.0–4.5 mg/dL
Protein, Total	5.3 g/dL	6.0 to 8.3 g/dL
albumin	2.5 g/dL	3.4–5.4 g/dL
Bilirubin, Total	1.5 mg/dL	0.1–1.2 mg/dL
ALT (SGPT)	16 U/L	7–55 U/L
AST	27 U/L	8–48 U/L
Alkaline Phosphatase	57 U/L	8–48 U/L
Magnesium	1.7 mg/dL	1.6–2.6 mg/dL
Lactate	0.75 mmol/L	0.7–2.1 mmol/L
Bilirubin, Indirect	1.2 mg/dL	0.3–1.0 mg/dL
C-Reactive Protein	7.7 mg/L	≤0.8 mg/dL
Procalcitonin	14.07 ng/mL	≤0.10 ng/mL

Malaria rapid antigen testing showed Plasmodium ovale, and the patient was started on artemether-lumefantrine. Preliminary blood culture reports suggested Gram-negative bacteremia, specifically Bacillus species (Cellulomonas spp/Microbacterium spp). However, subsequent analysis revealed that the result was a false positive, with the correct identification being Gram-positive and indicative of contamination. As a result, the antibiotics were discontinued. A second set of blood cultures, drawn minutes apart, returned negative. The peripheral blood smear confirmed Plasmodium ovale with a parasitemia level of 2.5% (reducing to 1% after treatment with antimalarials), and screening for other parasites was negative. The patient’s condition improved significantly within 24 hours of initiating artemisinin combination therapy. After testing for G6PD enzyme assay as negative, primaquine was added to the treatment regimen at discharge to address the hypnozoites of Plasmodium ovale. The patient was discharged in stable condition with instructions to continue primaquine and follow up outpatient.

## Discussion

The Centers for Disease Control and Prevention (CDC) reported that there are 2,000 malaria cases per year in the U.S., with most cases being imported from endemic regions of the world [6]. There is a worldwide decrease in malaria cases between 2000 and 2019, but the burden has remained unchanged in Sub-Saharan Africa [7]. Although malaria is not endemic to the US, imported cases have been reported and the vector of malaria is present in the U.S. which imposes a risk of transmission, making it a potential hotspot for the disease. There also have been a few U.S. cases linked to infected blood transfusions [8].

Among the various Plasmodium species, P. ovale has a relatively low prevalence, accounting for 0.5% to 10.5% of malaria cases, and only 3% of severe malaria cases [9,10]. In Sub-Saharan Africa, including Chad (P. ovale prevalence is 6.4%) [11] from where this patient traveled, the overall malaria burden has remained steady and P. ovale cases are often underreported due to partial immunity developed by people from repeated mosquito exposure, reducing infection severity, and due to difficulties in diagnosis using conventional methods such as rapid diagnostic tests (RDT) and microscopy. Because RDTs are not sensitive to detecting low-density parasitemia of P. ovale which is found in most people living in endemic areas, and morphologically it's difficult to distinguish between P. ovale and P. vivax [10,12,13]. The best tool for diagnosing P. ovale is polymerase chain reaction (PCR), which may not be readily available in endemic areas, takes longer than other common means of testing and is costly. Also, per CDC guidelines, repeat blood smears are required to confirm diagnosis if RDT is positive for P. ovale [14]. Correct diagnosis of malaria is crucial as delay in diagnosis and appropriate treatment is the leading cause of death from malaria in the U.S. D'Abramo et al. reported a case of imported malaria where a patient’s condition worsened to acute respiratory distress syndrome (ARDS) on oral chloroquine until proper treatment with IV artesunate and oral doxycycline was started [15]. The CDC suggests checking drug resistance in every malaria case in the U.S. to prevent delays in appropriate diagnosis and improve outcomes [6].

Severe malaria cases due to P. ovale have been reported with complications such as pulmonary impairment, hypotension/shock, bleeding/DIC, severe anemia, cerebral malaria, and jaundice [10]. According to a systematic review, jaundice, anemia, and pulmonary impairment are the most common complications. The mechanism of lung damage is explained as endothelial damage due to inflammation and parasitemia, which leads to capillary leak and interstitial edema. In the literature, the mortality rate from severe P. ovale is 30% from ARDS, 30% from splenic rupture, and 20% from acute renal failure (ARF) [16,17]. Risk factors for developing severe malaria include low immunity, pregnancy, hemoglobinopathies, and children. Despite the absence of these risk factors, cases of severe malaria from P. ovale have been reported in healthy pregnant adult travelers from endemic areas.

The treatment of malaria depends on the severity, malaria species, the part of the world it was acquired from, age, weight, and pregnancy. The treatment regimen and duration for malaria from different Plasmodium species are discussed here [18]. For severe malaria, the guidelines suggest parenteral artesunate or IV quinine. Interim treatment with parenteral artemether/lumefantrine or atovaquone/proguanil can be given. To prevent relapse from P. ovale, treatment with primaquine or tafenoquine for 14 days is recommended [6].

## Conclusions

This case illustrates very clearly the importance of imported malaria, particularly Plasmodium ovale infection, in a patient with a recent history of travel to an endemic area, even when no usual risk factors exist for the development of severe malaria. The rapid clinical improvement of the patient after the introduction of artemisinin combination therapy illustrates the importance of timely and appropriate treatment. At the same time, the initial diagnostic difficulty and possibly serious complications suggest the need for increased clinical awareness among healthcare workers in the US. The case also highlights the broader impact of malaria on public health in non-endemic regions, such as the United States. This is particularly true for imported cases that may pose a risk of transmission. Prompt and accurate diagnosis with adherence to guidelines for treatment, along with high suspicion for drug resistance, is important for improved outcomes and prevention of fatality in such cases.
